# BBMRI catalogue

**DOI:** 10.1186/2043-9113-5-S1-S19

**Published:** 2015-05-22

**Authors:** Klaus Kuhn, Raffael Bild, Helmut Spengler

**Affiliations:** 1 Chair of Medical Informatics, Technische Universität München, 81675 Munich, Germany

## Characterisation

Biobanks, access to biosamples, BBMRI.

## Description

**Figure 1:**
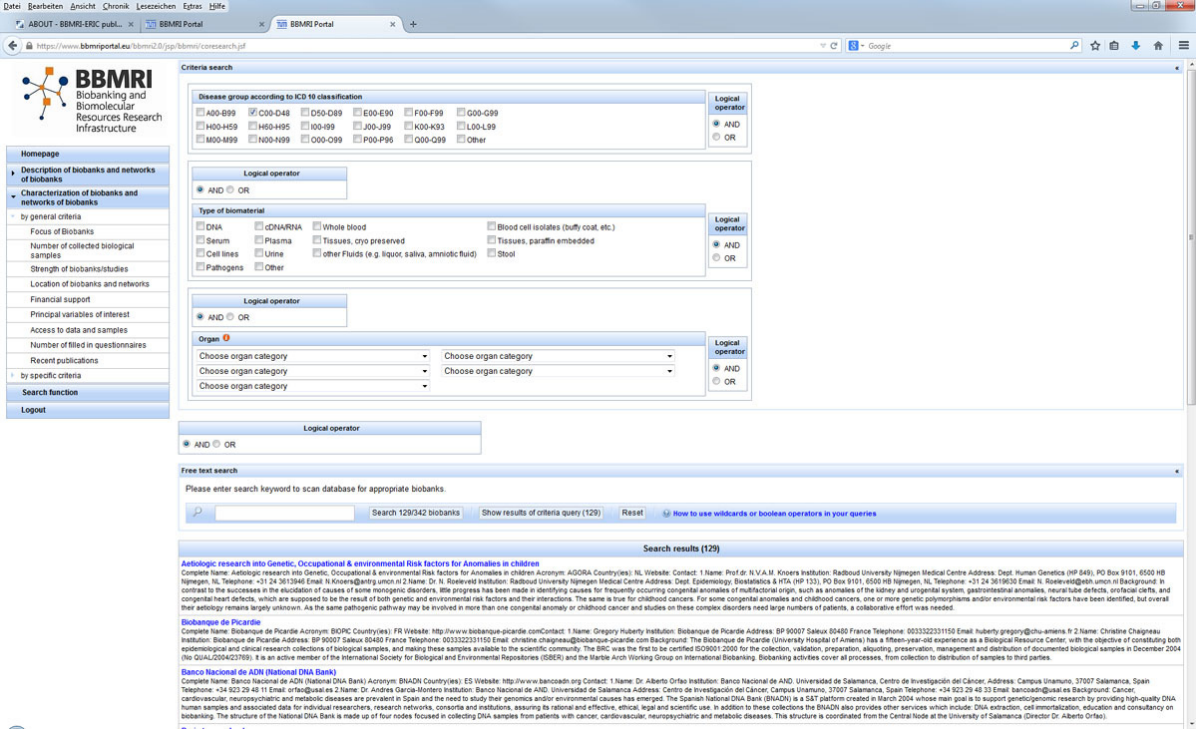
User interface of the BBMRI Catalogue displaying search functions and search results (text below)

During the preparatory phase of BBMRI (the European Biobanking and BioMolecular Resources Research Infrastructure), an online catalogue has been established for the collection and presentation of data describing the majority of European biobanks [1]. This catalogue can be publicly accessed at the web site bbmri.eu (select catalogue and then login as guest). Currently, it contains data from 330 biobanks located in 29 European countries. It can be browsed by different categories, and it contains contact data for each biobank (Figure 1). Browsing options include the numbers of biosamples per biobank and per material type, descriptions of diagnosis groups per biobank, and characterizations of access to data and samples. A search function is provided, allowing the selection of criteria (e.g. according to disease group, type of biomaterial, category of organ, combinations of all these). Figure 1 illustrates these functions. The catalogue is being further developed, with a focus on compatibility to MIABIS [2] and on a data warehouse layer.

## Status of development

Operational since 2011. It is being further developed in BBMRI-ERIC.

## Users

Scientists, biobank researchers, clinical investigators.

## Links

http://bbmri.eu, http://bbmri-eric.eu
